# Rituximab and intravenous immunoglobulin treatment in PM/Scl antibody-associated disease: case-based review

**DOI:** 10.1007/s00296-021-05075-z

**Published:** 2022-01-10

**Authors:** Denesh Srikantharajah, Mark E. Lloyd, Patrick D. W. Kiely

**Affiliations:** 1 Department of Rheumatology, St George’s University Hospitals NHS Foundation Trust, London, SW17 0QT UK; 2 Department of Rheumatology, Frimley Healthcare NHS Foundation Trust, Frimley, UK; 3 Institute of Medical and Biomedical Education, St George’s, University of London, London, UK

**Keywords:** PM/Scl autoantibody, Myositis, Systemic sclerosis, Raynaud’s, Rituximab, Intravenous immunoglobulin

## Abstract

Autoantibodies to the 75-kDa and 100-kDa subunits of the PM/Scl nucleolar protein complex are associated with an overlap syndrome, manifesting with clinical features of systemic sclerosis and idiopathic inflammatory myopathy. We describe the diverse clinical features in a series of 4 cases with anti-PM/Scl-75 and/or anti-PM/Scl-100 antibodies, including severe proximal muscle weakness, oesophageal dysfunction, respiratory weakness requiring mechanical ventilation, Raynaud’s, calcinosis cutis, sclerodactyly and critical digital ischaemia. Despite the severity of striated and oesophageal muscle weakness, all patients responded very well to immune suppression, and calcinosis cutis in one case regressed substantially. We highlight the efficacy of Rituximab and intravenous immunoglobulin therapy (IVIg) in these cases, enabling return to normal muscle function within six months. Rituximab was preferentially chosen for cases with hyper-gammaglobulinemia and multiple autoantibodies in addition to anti-PM/Scl, and IVIg was utilised for cases where a rapid onset of effect was required, such as severe ventilator-dependent respiratory muscle weakness and oesophageal dysfunction.

## Introduction

We present a case series based on four patients with anti-PM/Scl-75 and/or anti-PM/Scl-100 antibodies. Our aim was to assess the efficacy and tolerability of Rituximab or intravenous immunoglobulin (IVIg) therapy in these patients with diverse clinical features of anti-PM/Scl 75/00-associated disease. The clinical and serologic data were collated retrospectively, from case note and hospital record review at St George’s University Hospitals and Frimley Healthcare NHS Foundation Trusts, UK. All patients provided verbal informed consent for their details and images to be included in this case series. Ethic approval was not required. Antibodies were measured using immunoblot and processed by the Immunology laboratory Royal United Hospital, Bath and the Protein Reference Unit, Northern General Hospital, Sheffield. A summary of demographics, clinical features, serology and immunosuppressive therapies is shown in Table 1.

**Table 1:** Summary of demographic, clinical, serologic features, and treatments in four cases with anti-PM/Scl antibodies

	Case 1	Case 2	Case 3	Case 4
Gender / Age	M / 36	M / 19	F / 42	F / 42
PM/Scl 75/100	75, weak 100	75 / 100	100	100
ANA titre ENA Polyclonal Ig	160–640 +	1280–2560 SSA, SSB, dsDNA **+ **	80	1280
Striated myopathy Peak CK (U/L)	+ 8120	+ 3008	+ 3484	+ 1364
Oesophageal dysfunction	+ +	+	+ +	+ +
Respiratory muscle			+	+ +
Weight loss	+ +	+	+ +	+ +
Raynaud’s		+	+	+
Sclerodactyly		+ +	+	+
Microstomia		+	+	+
Mechanics hands			+	
Calcinosis cutis		+		
Arthritis		+	+	
Initial therapy	P, Aza	P, MMF	IVMP, P, Aza	IVH, P, MMF/Aza
Additional therapy	Rituximab	Rituximab	IVIg	IVIg

Key. *SSA/B* Sjogren’s syndrome A/B (Ro/La) autoantibody, *dsDNA* double-stranded DNA autoantibody, *P* prednisolone, *Aza* azathioprine, *MMF* mycophenolate mofetil, *IVMP* intravenous methyl prednisolone, *IVH* intravenous hydrocortisone, *IVIg* intravenous immunoglobulin

## Case 1 Anti-PM/Scl 75, weak anti-PM/Scl-100

### Myopathy with oesophageal involvement

A 36-year-old gentleman of black ethnic origin presented with a 10-week history of myalgia with rapidly progressive weakness, dysphagia to liquids and solids with nasal regurgitation, nasal speech and 12 kg weight loss. He also reported arthralgia and non-pleuritic chest pain. There were no preceding viral symptoms.

On examination, there was symmetric lower limb proximal weakness, hip flexor MRC grade 3 + /5, and inability to stand from a chair with arms folded. Walking was limited by weakness to a few paces. Upper limb and neck flexor strength were normal. There were no cutaneous features of dermatomyositis or systemic sclerosis, nor joint swelling. Cardiorespiratory and abdominal examination were normal, with the exception of high blood pressure.

**Fig. 1:**
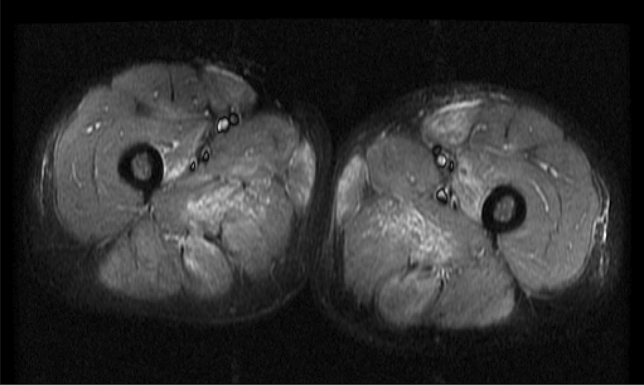
Case 1. Fat-suppressed T2 MRI thighs, showing increased signal in adductor magnus, gracilis, vastus intermedius and rectus femoris muscles

Serum creatine kinase (CK) was 8120 U/L, and 25OH vitamin D 27 nmol/L. Serology showed ANA 1/160–1/640 (speckled, nucleolar staining) with positive anti-PM/Scl 75 and weak anti-PM/Scl-100, but negative ENA, RF and myositis-specific antibodies and normal complement C3 and C4. Total IgG was elevated (17.2 g/L) with no paraprotein. Serology for hepatitis B, C and HIV was negative. MRI thighs confirmed bilateral symmetrical oedema in the gluteal and thigh muscles, including rectus femoris, adductor magnus, gracilis, sartorius and tensor fasciae latae (Fig. 1). Muscle biopsy from sartorius showed features of a necrotising inflammatory myopathy with a lymphocytic and macrophage infiltrate.

He was treated with prednisolone 1 mg/kg body weight, and azathioprine 1.25 mg/kg body weight. After three weeks, he reported a partial response in dysphagia and strength, was able to do 12 chair stands with folded arms in 30 s, and CK reduced to 3886 U/L. Given the antibody profile and hypergammaglobulinemia, Rituximab was commenced with an initial 2 × 1 g dose, and Azathioprine was increased to 2 mg/kg body weight. Six weeks later, he reported that dysphagia had completely resolved, and lower limb strength improved to grade 4 + /5 hip flexion, with the ability to do 14 chair stands with folded arms in 30 s. Global strength, on a visual analogue scale, improved from 1/10 to 8/10 and exercise endurance increased from a few paces pre-treatment to 30 min without rest.

Within 3 months of Rituximab, he felt subjectively back to normal strength and swallow. Serum CK fell to 939 U/L by month 6, possibly remaining out of normal range given his black ethnicity. Remission was maintained with Rituximab 1 g every 6 months, azathioprine 2 mg/kg and prednisolone 5 mg daily was tapered.

## Case 2 Anti-PM/Scl 75, anti-PM/Scl-100

### Myopathy, oesophageal involvement, systemic sclerosis with calcinosis cutis

A 19-year-old man of white ethnic origin presented with gradual onset of several months upper and lower limb proximal weakness, difficulty climbing stairs, new Raynaud’s phenomenon, small and large joint arthralgia and knee swelling. He also reported mild dyspnoea, mild dysphagia without choking or regurgitation, and mild weight loss.

**Fig. 2:**
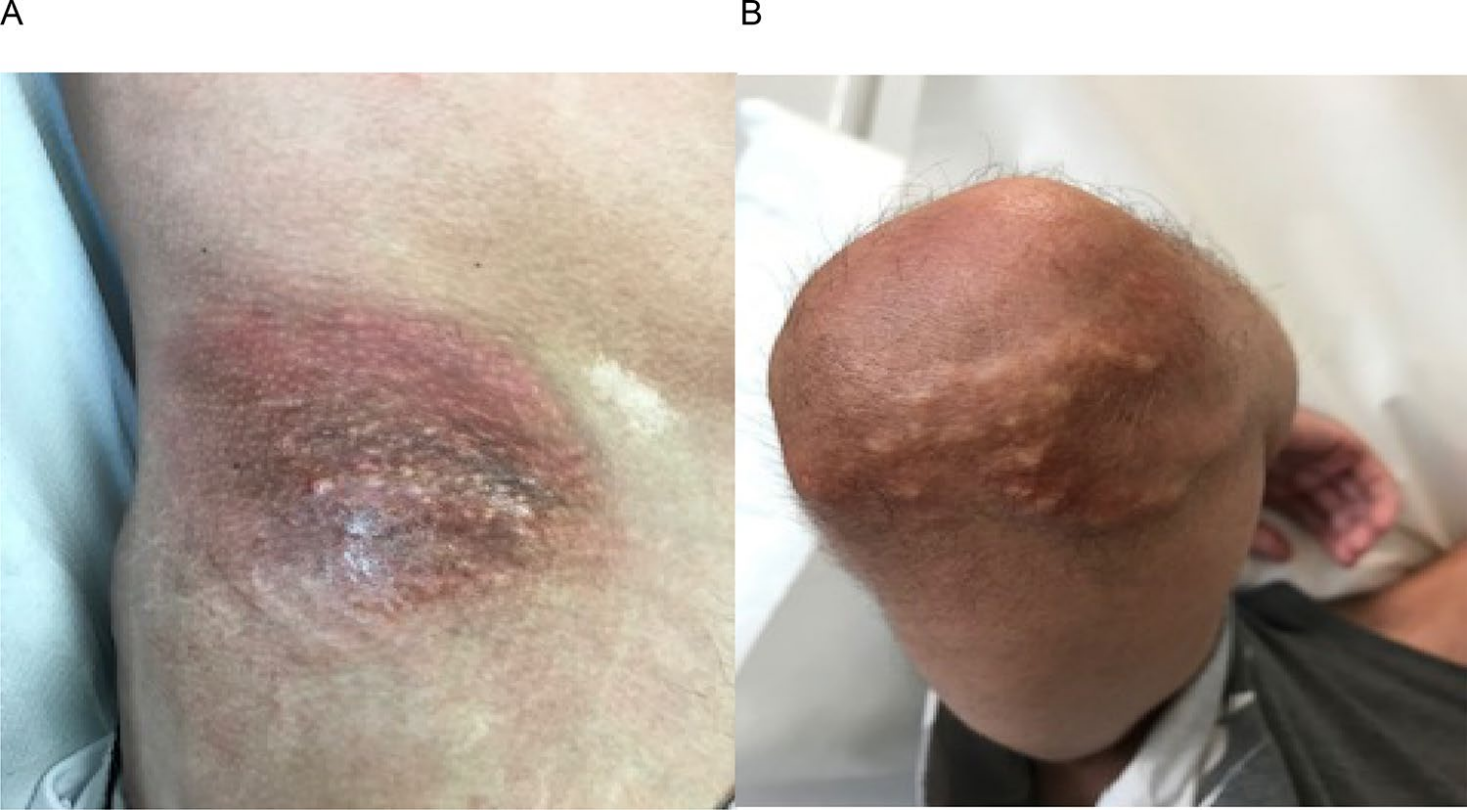
Case 2. Image of calcinosis cutis at the anterior iliac crest (**A**) and right elbow (**B**)

On examination, there was microstomia, sclerodactyly, sclerodermatous changes affecting the forearms, and ulcers involving the left third and right fourth and fifth digits. There were plaques of calcinosis cutis at the extensor surface of the elbows and knees, anterior iliac crests and buttocks (Fig. 2). He was unable to stand from a chair with arms folded. There was proximal weakness in upper and lower limbs, MRC grade 4/5 power, and preserved distal power.

Serum CK was 3008 U/L and serology showed ANA 1/1280–1/2560 (mixed homogeneous and nucleolar pattern), with positive anti-PM/Scl 75 and anti-PM/Scl-100, dsDNA 71 (Crithidia negative), positive SSA and borderline SSB and negative myositis-specific antibodies. There was a polyclonal hyper-gammaglobulinemia, IgG 28.1 g/L, low C3 (0.7) and C4 (0.13), and 25OH vitamin D was deplete, 18 nmol/L.

He was treated with prednisolone 20 mg daily, mycophenolate mofetil (MMF) 1 g twice daily and Tadalafil for ischaemic skin ulcers. At three months, with a partial response in strength, MMF was optimised to 1.5 g twice daily and Rituximab was commenced in view of a strong antibody profile and hyper-gammaglobulinemia, with an initial 2 × 1 g dose, and repeated 1 g × 4–6 monthly. For calcinosis cutis, he received colchicine 500 mcg twice daily and diltiazem 240 mg twice daily.

Following two cycles of Rituximab, his strength recovered with the ability to do 14 chair stands with folded arms in 30 s, and global strength improved from 2/10 to 7.5/10. His exercise tolerance increased from walking for a maximum of five minutes pre-treatment to one hour without rest. He re-gained normal weight with resolution of dysphagia. Serum CK normalised to 96 U/L four months after commencing Rituximab. Raynaud-associated ischaemic skin lesions all healed promptly with Tadalafil. Calcinosis cutis lesions became less tender, and regressed substantially over 12 months.

## Case 3 Anti-PM/Scl-100

### Myopathy, oesophageal and intercostal muscle involvement, systemic sclerosis

A 42-year-old woman of white ethnic origin presented with 10 months of progressive upper and lower limb weakness with falls, dysphagia to liquids and solids with choking and 10 kg weight loss. She also reported hand puffiness and new Raynaud’s phenomenon.

**Fig. 3:**
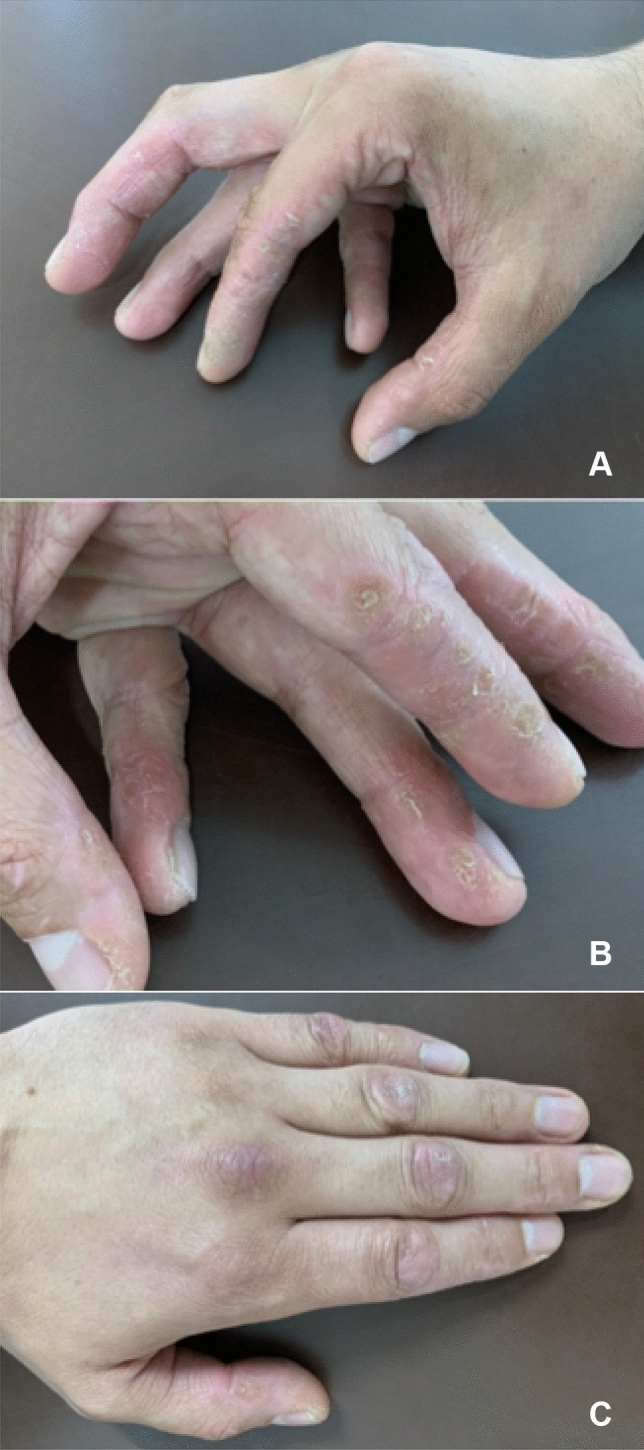
Image of extensive Mechanic’s hands (**A**, **B**) and Gottron’s papules (**C**) seen in anti-PM/Scl antibody disease

On examination, there was proximal weakness in upper and lower limbs, MRC grade 4/5, inability to stand from a chair with folded arms, and preserved neck flexor and distal limb power. There were features of microstomia, sclerodactyly, and mechanics hands with dry cracked hyperkeratotic skin on the radial aspect of the fingers (Fig. 3). Cardiorespiratory and abdominal examination were normal.

Serum CK was 3484 U/L, with elevated TSH 9.11 and normal 25OH vitamin D 122 nmol/L. Serology showed ANA 1/80 (homogeneous and nucleolar staining, chromosome-positive) with positive anti-PM/Scl-100, but negative ENA, RF and myositis-specific antibodies. Immunoglobulins and complement C3 and C4 were normal. MRI thighs showed oedema in the muscles of the pelvis and both anterior and posterior compartments of the thighs, accompanied by peri-fascial oedema. Muscle biopsy revealed a necrotising inflammatory myopathy with a predominantly lymphocytic infiltrate.

She was treated with intravenous methylprednisolone, oral prednisolone 1 mg/kg body weight and Azathioprine up to 2.5 mg/kg body weight. At eight weeks, IVIG (2 g/kg body weight) was commenced in view of severe dysphagia and repeated every six weeks, for a total of six cycles. She required a CPAP machine for respiratory support whilst asleep.

Within a month of IVIg, serum CK normalised, and at three months, she had recovered normal swallow, no longer required CPAP support at night and strength improved rapidly. Exercise endurance increased from walking a maximum of two minutes to 2 h without rest. After 6 months, prednisolone was discontinued and remission was maintained with azathioprine 2 mg/kg body weight.

## Case 4. Anti-PM/Scl-100

### Myopathy, oesophageal and severe intercostal muscle involvement, systemic sclerosis

A 42-year-old woman of white ethnic origin presented with a year’s history of gradually increasing weakness, dysphagia with food regurgitation, 8 kg weight loss, exertional dyspnoea and new Raynaud’s phenomenon.

On examination, there was marked truncal weakness and MRC grade 4/5 proximal upper and lower limb weakness. There were features of microstomia and sclerodactyly with dilated nail fold capillaries. She was hypoxic on room air with a respiratory acidosis (pH 7.2, pCO2 9.82, pO2 9.02) and lung auscultation was normal. Serum CK was 1364 U/L, ANA 1:1280 (nucleolar), PM/Scl-100-positive, negative PM/Scl-75, RF, ENA, dsDNA and myositis-specific antibodies.

In view of severe respiratory weakness, she required assisted ventilation, failed non-invasive nasal ventilation and was intubated and ventilated on intensive care. She was initially treated with IVIg (2 g/kg body weight), methyl prednisolone and intravenous hydrocortisone whilst ventilated for 3 weeks. This was followed by oral prednisolone 20 mg and MMF 1 g twice daily. She received a second course of IVIg after 5 weeks and MMF was switched to azathioprine 2 mg/kg body weight.

She gradually improved and was discharged 7 weeks after admission, self-caring, though still requiring pureed food. After 6 months, she had regained normal weight and swallow and was fully independent. CK reduced quickly after IVIg treatment and was within normal range within 4 months. Maintenance of remission with azathioprine and tapering prednisolone was continued.

## Discussion

Anti-PM/Scl autoantibodies are associated with a varied phenotype, including clinical features classically associated with idiopathic inflammatory myopathies (IIM) and systemic sclerosis (SSc). As well as myopathy, patients may have arthritis, Raynaud’s syndrome, dysphagia, oesophageal reflux, interstitial lung disease, telangiectasia, puffy hands, subcutaneous oedema, sclerodactyly, calcinosis cutis, heliotrope rash, mechanic’s hands and Gottron’s papules or sign. Sicca symptoms and primary Sjogren’s syndrome are also reported [1–3]. The prevalence of symptoms in case series varies widely, especially renal involvement reported in 0–21% [3, 4]. Whilst this could partly be explained by small sampling bias, it reflects the variable phenotype associated with these autoantibodies.

Overall, the four cases presented in this series demonstrate the diverse features of anti-PM/Scl autoantibodies. The ages ranged from 19 to 42, of both genders, a mix of ethnicities and a constellation of symptoms and signs spanning the features of IIM and SSc. Significant weight loss (8–12 kg), limb muscle weakness, with peak CK ranging from 1364 to 8120 U/L, and oesophageal involvement were found in all cases. Intercostal respiratory muscle weakness was a feature of cases 3 and 4 requiring assisted ventilation. Dermatological manifestations were absent in Case 1, and ranged from Mechanic’s hands in Case 3 to new onset Raynaud’s phenomenon, sclerodactyly and microstomia in Cases 2,3, and 4 and more generalised features of systemic sclerosis with ischaemic digital ulcers and extensive calcinosis cutis in Case 2. No cases had interstitial lung disease at presentation, though when described, it is noted to be generally mild and non-aggressive compared to patients with Scl-70 antibodies and the anti-synthetase syndrome (ASS) [1, 3, 5].

A study of 41 patients with anti-PM/Scl autoantibodies compared the prevalence of a wide range of potential features with comparator groups of patients with ASS, dermatomyositis (DM) and immune-mediated necrotizing myopathy (IMNM) [1]. This concluded that weakness was very prevalent in patients with anti-PM/Scl autoantibodies, in a distinctive pattern whereby arm abductors were weaker than hip flexors, not seen in DM, ASS and IMNM [1]. Moreover, this study reported a higher prevalence of mechanics hands, Raynaud’s and calcinosis in anti-PM/Scl 75/100 patients compared to ASS, DM and IMNM cases. These features were all present in our series, though the specific pattern of weakness proposed was not seen.

It is unclear whether patients with autoantibodies to PM/Scl-75 alone, and PM/Scl-100 alone have different clinical manifestations to those with both autoantibodies. Whilst some studies have indicated single positivity of each associates with discrete clinical subsets [6], others have found no differences [1]. Cancer has been reported amongst SSc patients to be associated with anti-PM/Scl 100 alone, and not anti-PM/Scl 75 alone or both reactivities [7]. In our series, 2 patients were single positive. Cases 3 and 4 (anti-PM/Scl-100 alone) were noteworthy for having severe intercostal muscle involvement not seen in the others. In contrast, in Cases 1 and 2 (anti-PM/Scl-75 and anti-PM/Scl-100), there were no unique features. The phenotypes of Cases 2, 3, and 4 were similar with IIM and cutaneous features of SSc dominating, whereas case 1 had less extensive disease, restricted to the IIM spectrum.

Previously published literature has not focused on the use of Rituximab and IVIg. In this case series, we highlight the effectiveness of both treatments. Strikingly, in all cases, an excellent clinical and biochemical response to immunosuppression was seen. Whilst steroids enabled a rapid decline in the CK levels, the addition of Rituximab or IVIg ensured continued clinical and biochemical improvement, with full recovery of striated and oesophageal muscle function within six months of treatment. Optimising muscle function may also require correction of hypovitaminosis D, as was necessary in 2 cases. It is also noteworthy that the extensive calcinosis cutis in Case 2, which was additionally treated with diltiazem and colchicine, demonstrated remarkable regression over a longer time frame.

The rationale for starting Rituximab in Cases 1 and 2 was the presence of hyper-gammaglobulinemia and strong autoantibody profiles (ANA, SSA, SSB and dsDNA). In contrast, in Cases 3 and 4, IVIg was chosen because of severe oesophageal and intercostal muscle involvement and the need for a fast therapeutic effect. A recent retrospective analysis indicated that the addition of IVIg is effective for refractory dysphagia associated with IIM [8, and our two cases confirm this benefit for both oesophageal and intercostal respiratory muscle disease. The quicker onset of action of IVIg in comparison to Rituximab was particularly needed in Case 4, who required mechanical ventilation.

In conclusion, patients with anti-PM/Scl 75/100 antibodies exhibit a diverse range of clinical features encompassing classic IIM and SSc. We highlight that despite severe manifestations, outcome to immune suppression is favourable and Rituximab and IVIg are highly effective treatment strategies. Our series reflects the utility of clinical phenotypes and antibody profiles to choose appropriate immunosuppressive agents, with IVIg favoured for oesophageal and critical intercostal involvement where a rapid onset of effect is desirable. Rituximab is a logical choice for any B cell-driven disease characterised by autoantibodies and hyper-gammaglobulinemia, and we demonstrate that this equally applies to patients with anti-PM/Scl disease. Nonetheless, our series is small and it would be useful to expand these findings to larger randomised controlled trials to confirm the efficacy reported here.
